# Characteristics of land-atmosphere interaction parameters in hinterland of the Taklimakan Desert

**DOI:** 10.1038/s41598-020-66029-2

**Published:** 2020-06-09

**Authors:** Yongqiang Liu, Xianyong Meng, Ali Mamtimin, Qing He

**Affiliations:** 1 College of Resources and Environmental Sciences, Xinjiang University, Urumqi, 830046 China; 2 Key Laboratory of Oasis Ecology, Ministry of Education, Xinjiang University, Urumqi, 830046 China; 3 College of Resources and Environmental Sciences, China Agricultural University (CAU), Beijing, 100094 China; 4 Department of Civil Engineering, The University of Hong Kong (HKU), Pokfulam, 999077 Hong Kong, China; 5 Taklimakan Desert Meteorology Field Experiment Station of CMA, Institute of Desert Meteorology, China Meteorological Administration, Urumqi, 830002 China

**Keywords:** Atmospheric dynamics, Hydrology, Hydrology

## Abstract

The importance of the energy exchange between the land surface and the atmosphere can be characterized by bulk transfer coefficients for momentum, *C*_*d*_, and heat, *C*_*h*_. The diurnal and monthly variations of both bulk transfer coefficients and lengths of surface roughness are analyzed. Based on observed data from January to December 2009 in hinterland of the Taklimakan Desert, the characteristics of aerodynamic roughness length, *z*_*0m*_, and thermal roughness length, *z*_*0h*_, are discussed. It should be noted that the diurnal and monthly variations of the parameters are fundamentally different from those reported in vegetated areas. Specifically, four unique features can be identified in the surface layer. First, in Taklimakan Desert, *z*_*0m*_ does not vary with seasons; however, it significantly depends on wind speed. Second, *z*_*0h*_ is higher in the daytime and lower at night, showing obvious diurnal characteristics. The high values appear at sunrise and sunset. Third, both *C*_*d*_ and *C*_*h*_ have two peaks, one peak at sunrise, and another one at noon. Fourth, both *C*_*d*_ and *C*_*h*_ have larger values in winter season and smaller values in summer season.

## Introduction

The vast Taklimakan Desert (TD) significantly influences the climate formation in Northwestern China^1–4^, and the energy exchange at the interface of land and the atmosphere drives the climate system^5,6^. The energy exchange is dependent on several important parameters, including the surface albedo (*α*), the surface emissivity (ε), the aerodynamic and thermal roughness lengths (*z*_0*m*_ and *z*_0*h*_), and the bulk transfer coefficients for momentum *C*_*d*_ and heat *C*_*h*_. Thus, to study these parameters is of great importance. Many field experiments, such as HAPEX/MOBILMY^7^, FIFE^8^, HEIFE^9^, EFEDA^10^, BOREAS^11^, IMGRASS^12^, NOPEX^13^, GAME^14^, NWC-ALIEX^15^, EBEX-2000^16^, LOPEX^17^ and others, have focused on the interactions between the surface of land and the atmosphere. In the TD, many studies have been performed about the land surface key parameters, atmospheric boundary layer and its stability. Liu *et al*.^18^ calibrated some key parameters by *in-situ* observed data of the hinterland of TD, including the averaged surface albedo (*α*), surface emissivity (ε), soil thermal conductivity (*λ*_*s*_), and aerodynamic roughness lengths (*z*_0*m*_). In addition, several formulas or schemes for the thermal roughness length (*z*_0*h*_) in the common land model were assessed. Liu *et al*.^19^ investigated two completely different methods for the calculation of surface emissivity. Jin *et al*.^20^ analyzed the above important parameters (e.g., *α*, ε, *z*_0*m*_, *z*_0*h*_, *C*_*d*_ and *C*_*h*_) in the hinterland of TD, which was located in the northern edge of TD and spatially inhomogeneous. Aynigar *et al*.^21^ re-estimated the global terrestrial satellite broadband emissivity and moderate-resolution imaging spectrometer broadband emissivity in the TD regions. Wang *et al*.^22^ investigated the vertical structures of the convective boundary layer during the day and the stable boundary layer at night. In addition, they also explored the effects of sand-dust and rainfall events on the structure of the atmospheric boundary layer in the hinterland of TD in summer. On other deserts, Chen *et al*.^23^ assessed the significance of parameterizing *z*_0*h*_, and revealed that the revised *z*_0*h*_ scheme can successfully simulate the surface temperature and turbulent heat flux of arid regions in western China.

The fluxes of momentum and heat are two key parameters in the characterization of the energy exchange processes^24^, which are typically determined by the bulk transfer model and exchange coefficients (i.e., for both momentum *C*_*d*_ and heat *C*_*h*_)^25^. The bulk transfer coefficients are critical parameters controlling the total turbulent momentum and energy transported from the surface of land to the atmosphere. They directly reflect the land-atmosphere coupling strength. Thus, investigating their characteristics and variations is essential for calculating the energy exchange of land-atmosphere interactions.

**Figure 1:**
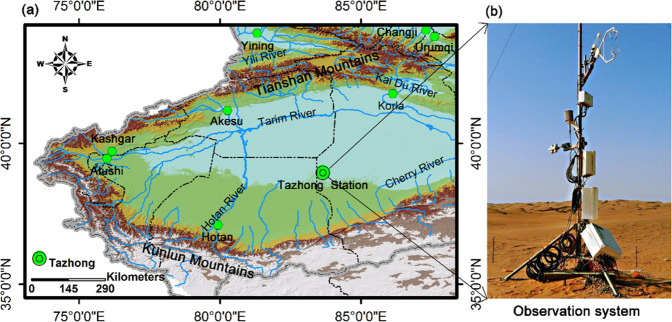
(**a**) Location of the Tazhong Station in Taklimakan Desert. (**b**) Eddy covariance measurement system and radiation observation system in the station. The map was generated using ArcMap Version 10.1 (http://www.esri.com/en/arcgis/arcgis-for-desktop/).

Taklimakan Desert is the driest area in China. It is also the second largest flow desert in the world, following the Sahara desert in Africa. Therefore, investigating *C*_*d*_ and *C*_*h*_ in the hinterland of Taklimakan Desert can reveal the physical processes governing the regional climates. The characteristic variations of *C*_*d*_ and *C*_*h*_ in the northern edge of TD have been investigated. However, in hinterland of the TD, *C*_*d*_ and *C*_*h*_ exhibit significant differences from other regions. This study defines *C*_*d*_ and *C*_*h*_, using data directly measured by the atmospheric environment observation station, located in Tazhong in the hinterland of the TD (Fig. 1, hereinafter Tazhong Station).

The defined coefficients can be applied to similar land surfaces where direct measurements are not possible or not practical. Prior to the process, two key parameters, i.e., aerodynamic roughness length (*z*_*0m*_) and thermal roughness length (*z*_*0h*_) should be determined. They are crucial parameters for calculating the turbulent flux via bulk transfer equations. The aerodynamic roughness length and thermal roughness length are defined as the surface nonslip condition and surface temperature, respectively, which can be applied in the framework of the Monin-Obukhov Similarity (MOS) theory. Based on the MOS theory, *z*_*0m*_ is the height at which the speed of the extrapolated wind vanishes, and *z*_*0h*_ is the height at which the temperature of the extrapolated air is equal to the surface temperature. Generally speaking, *z*_*0h*_ is significantly different from *z*_*0m*_ because the momentum transport is partly dependent on the turbulent resistance on roughness obstacles, whereas the heat transport is not related to these obstacles. *z*_*0h*_ refers to the parameterization of heat transport mechanisms near the surface, where the molecular viscosity and the molecular thermal diffusivity of air may have significant impacts on the heat transport^26^. The results from both experiments and theoretical analyses have demonstrated that *z*_*0m*_ ≠ *z*_*0h*_ in many cases^25,27^. Many published works^23,28–33^ have shown that *z*_*0h*_ is not constant. Instead, it has been demonstrated to have great diurnal and monthly variations.

## Results and Discussions

### Determination of aerodynamic roughness length

**Figure 2:**
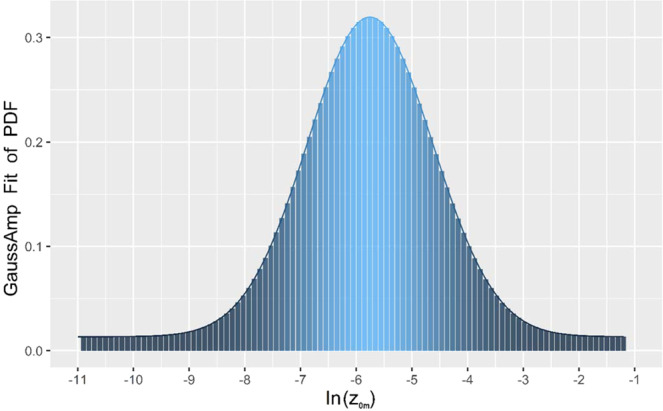
Distribution of ln(z_*0m*_) obtained by Eq. (2) using all data from 2009. The value of ln(z_*0m*_) was calculated to be −5.77 at the peak frequency in the curve, which was considered as the optimal value. The solid curve is fit for the histogram.

The continuously obtained data, which was recorded at 30 min intervals, were used to estimate *z*_*0m*_ in 2009. Due to the observation errors in the data or the fact that the meteorological conditions did not meet the similarity theory, the resulting ln(*z*_*0m*_) may be multiple values. Therefore, the distribution of ln(*z*_*0m*_) was calculated by SAS (Statistics Analysis System). The probability distribution function (PDF) of ln(*z*_*0m*_) was obtained. The value of *z*_*0m*_ at the peak frequency of the histogram of ln(*z*_*0m*_) was considered as the optimal value. Figure 2 shows at the peak frequency, ln(*z*_*0m*_) is −5.77. Thus, ln(*z*_*0m*_) = −5.77 and *z*_*0m*_ = 3.11 × 10^−3^ m.

**Figure 3:**
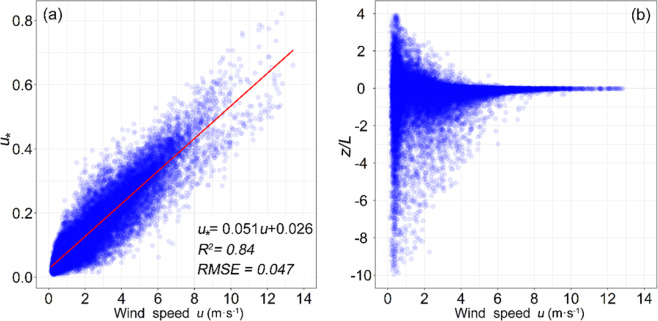
The relationship between the wind speed *u* with frictional velocity u_*_ (**a**) and atmospheric stability *z*/*L* (**b**). The solid red line represents the fitted regression line.

**Figure 4:**
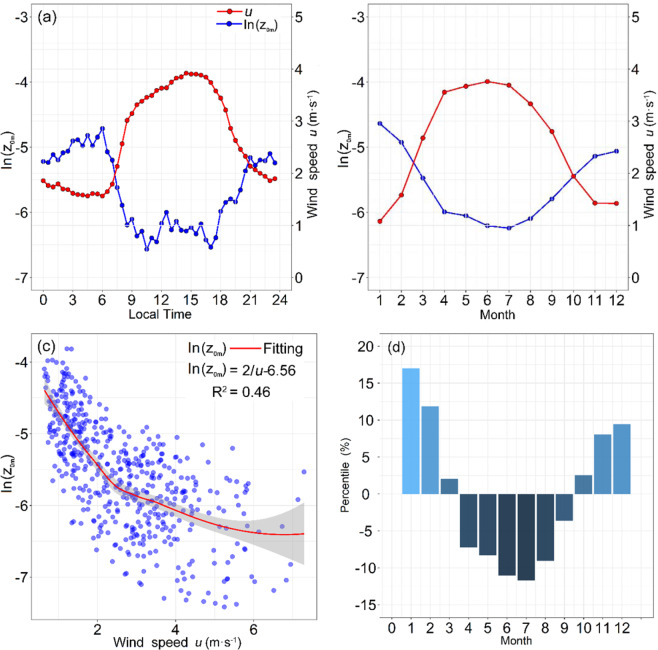
(**a**) Daily and (**b**) Monthly variations of the means of ln(*z*_*0m*_) and wind speed *u*. (**c**) Scatter plot of the daily mean of ln(*z*_*0m*_) and wind speed *u*, using all data from 2009. The solid red line represents the fitted curve. (**d**) Monthly changes in percentile relative to the annual mean.

In general, *z*_*0m*_ is a function of the vegetation that changes with the season. In addition, *z*_*0m*_ can also be affected by the speed of wind, as discussed in the following section ‘Surface roughness lengths’. However, the “seasonally varying vegetation” plays no role when analyzing a bare desert surface. Therefore, according to Eq. (2), the variation of *z*_*0m*_ is thus primarily related to *u*/u_*_ *and z*/*L*. Further analyses shown (Fig. 3) that the u_*_ and *z*/*L* are closely related to the wind speed. Liner regression equation, u* = 0.05 *u* + 0.026, indicates u_*_ linear dependence with *u*, and *z*/*L* tends to zero with wind speed increased. So, in the hinterland of TD, the Eq. (2) can be identified as: $\mathrm{ln}\,{z}_{0m}=\,\mathrm{ln}(z)-ku/(0.051u+0.026)-{\psi }_{m}(z/L)$. Hence, *z*_*0m*_ is primarily relate to wind speed. As indicated by the results in Fig. 4a,b, ln(*z*_*0m*_) is the reciprocal of *u*, but the integrated stability correction term for wind results in a time lag between the peaks of ln(*z*_0*m*_) and wind speed, on both diurnal and seasonal time scale. Thus, during the warm period from April 1 to September 30, *u* is stronger and *z*_*0m*_ is lower. During the cold period from October 1 to March 3, *u* is weaker and *z*_*0m*_ is higher. In addition, it varies monthly, from 1.95 × 10^−3^ m to 9.68 × 10^−3^ m; the variation is much smaller than those over vegetated regions. The monthly changes in percentile are shown relative to the annual mean 3.74 × 10^−3^ (Fig. 4d). Hence, the scatter plot of daily mean ln(*z*_*0m*_) and *u* demonstrates that ln(*z*_*0m*_) is inversely correlated with *u* (Fig. 4c). Their relationship can be fitted as:

1 $$\mathrm{ln}\,{z}_{0m}=2/u-6.56$$

### Determination of thermal roughness length

**Figure 5:**
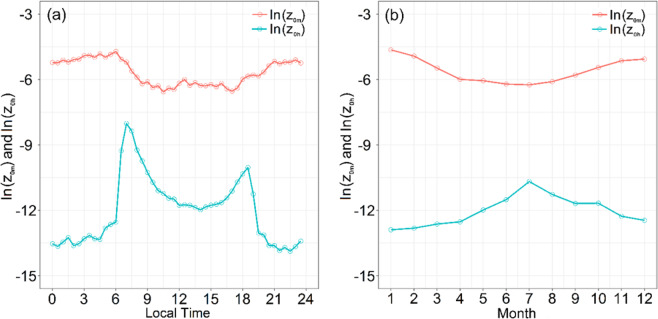
(**a**) Diurnal and (**b**) Monthly variations of the means of ln(z_*0m*_) and ln(z_*0h*_).

**Figure 6:**
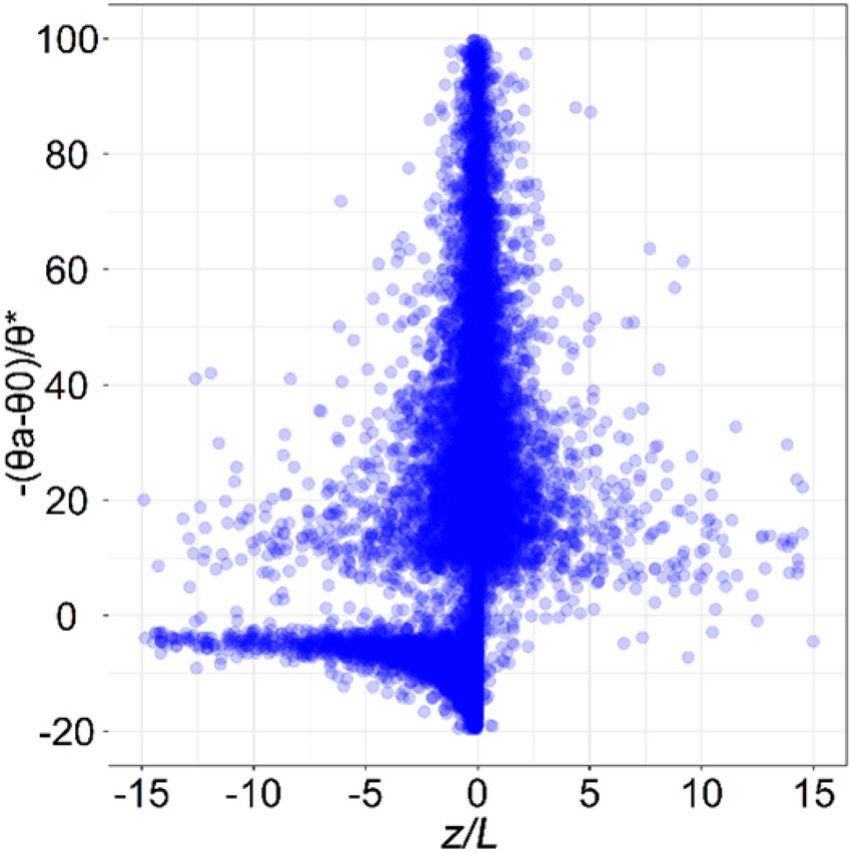
Diurnal variations of the −(*θ*_*a*_-*θ*_0_)/θ_*_ at different atmospheric stability (*z*/*L*) in all day, and close to peak in near-neutral atmospheric stratification.

Compare with *z*_*0m*_, *z*_*0h*_ displays more significant diurnal variations (Fig. 5a). The mean value of ln(*z*_*0h*_) in the daytime is considerably higher than at night, and the daytime variations are larger than those at night. *z*_*0h*_ exhibits diurnal variation, which is 9.78 × 10^−7^ m during the night time and 3.28 × 10^−4^ m in the daytime. Two peaks are observed for ln(*z*_*0h*_), i.e., one at sunrise and the other one at sunset. There is a slight trough between the two peaks. The two peaks occur at times when the atmospheric stability is near-neutral. In other words, the atmospheric stability will change from stable to unstable at sunrise, and change inversely at sunset. Under near-neutral conditions, the second term, –(*θ*_*a*_-*θ*_0_)/θ_*_, i.e., in Eq. (3), is close to peak (Fig. 6), while the third term *Ψ*_*h*_(*z*/*L*), which is the integral stability correction term of temperature profile, is close to zero. Therefore, ln(*z*_*0h*_) reaches maximum values at sunrise and sunset. In addition, ln(*z*_*0h*_) exhibits a greater monthly variation (Fig. 5b), with a peak occurring in July and a valley in January. It varies from 2.52 × 10^−6^ m to 2.31 × 10^−5^ m, with a mean of 7.93 × 10^−6^ m. Therefore, *z*_*0h*_ has a larger value in summer and a smaller value in winter. Since the variation of *z*_*0h*_ in the daytime is considerably higher than at night, and the daytime in summer is longer than in winter, accordingly, which result in the peak values occurred in July and valley in January.

### Determination of bulk transfer coefficients

**Figure 7:**
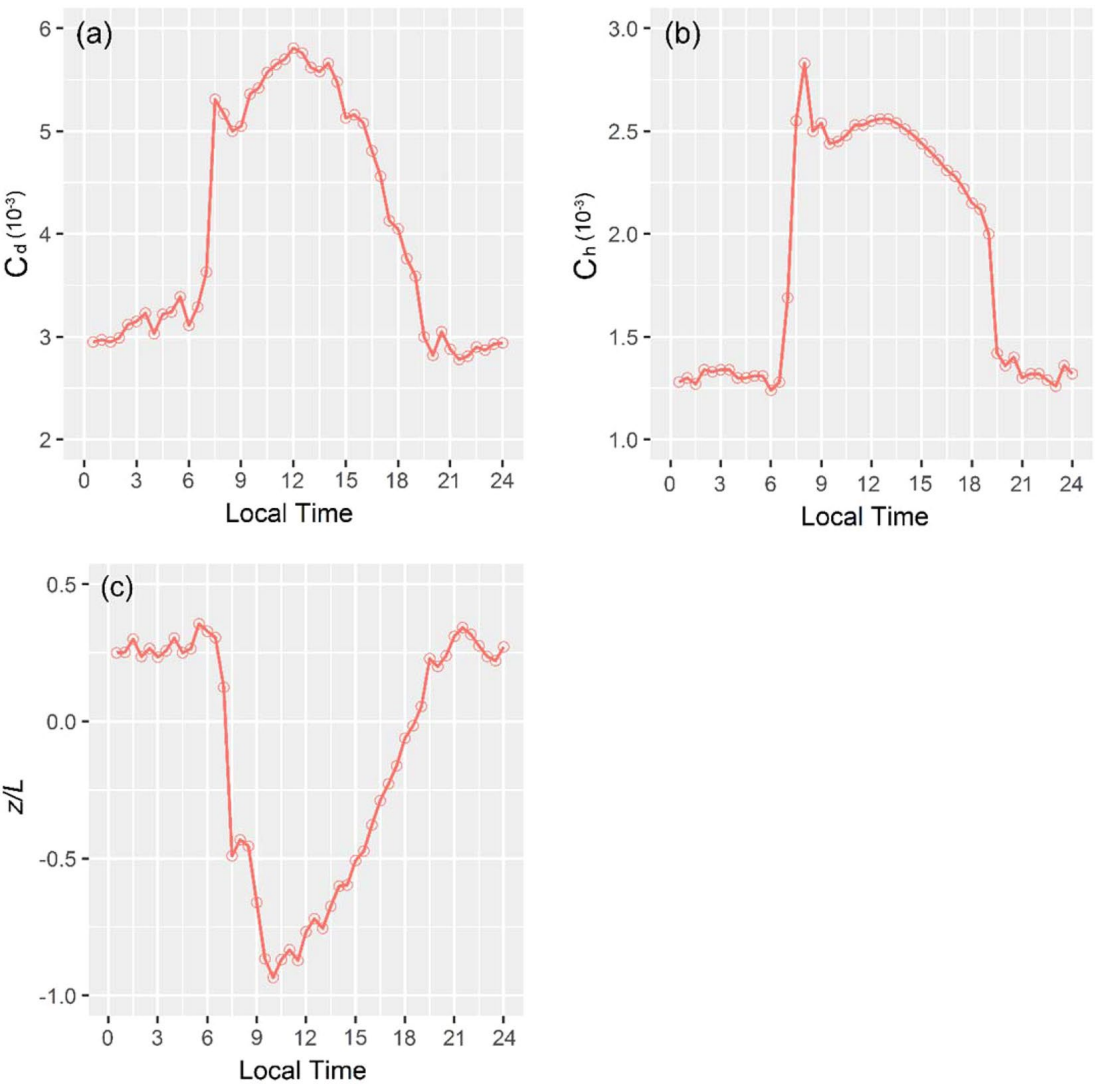
Diurnal variations of the means of (**a**) *C*_*d*_, (**b**) *C*_*h*_ and (**c**) atmospheric stability *z*/*L*.

**Figure 8:**
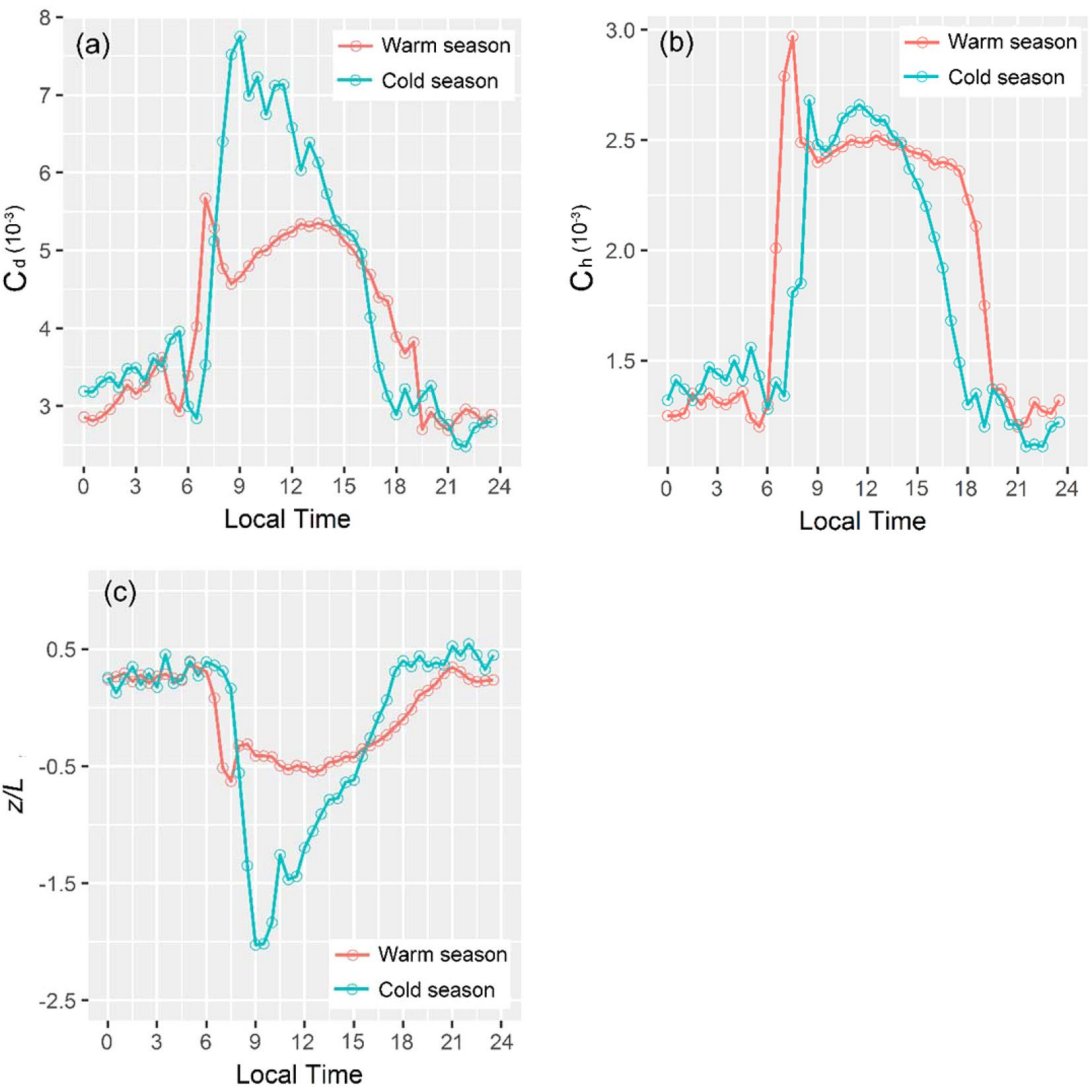
Diurnal variations of the means of (**a**) *C*_*d*_, (**b**) *C*_*h*_ and (**c**) atmospheric stability *z*/*L* in the cold and warm seasons.

**Figure 9:**
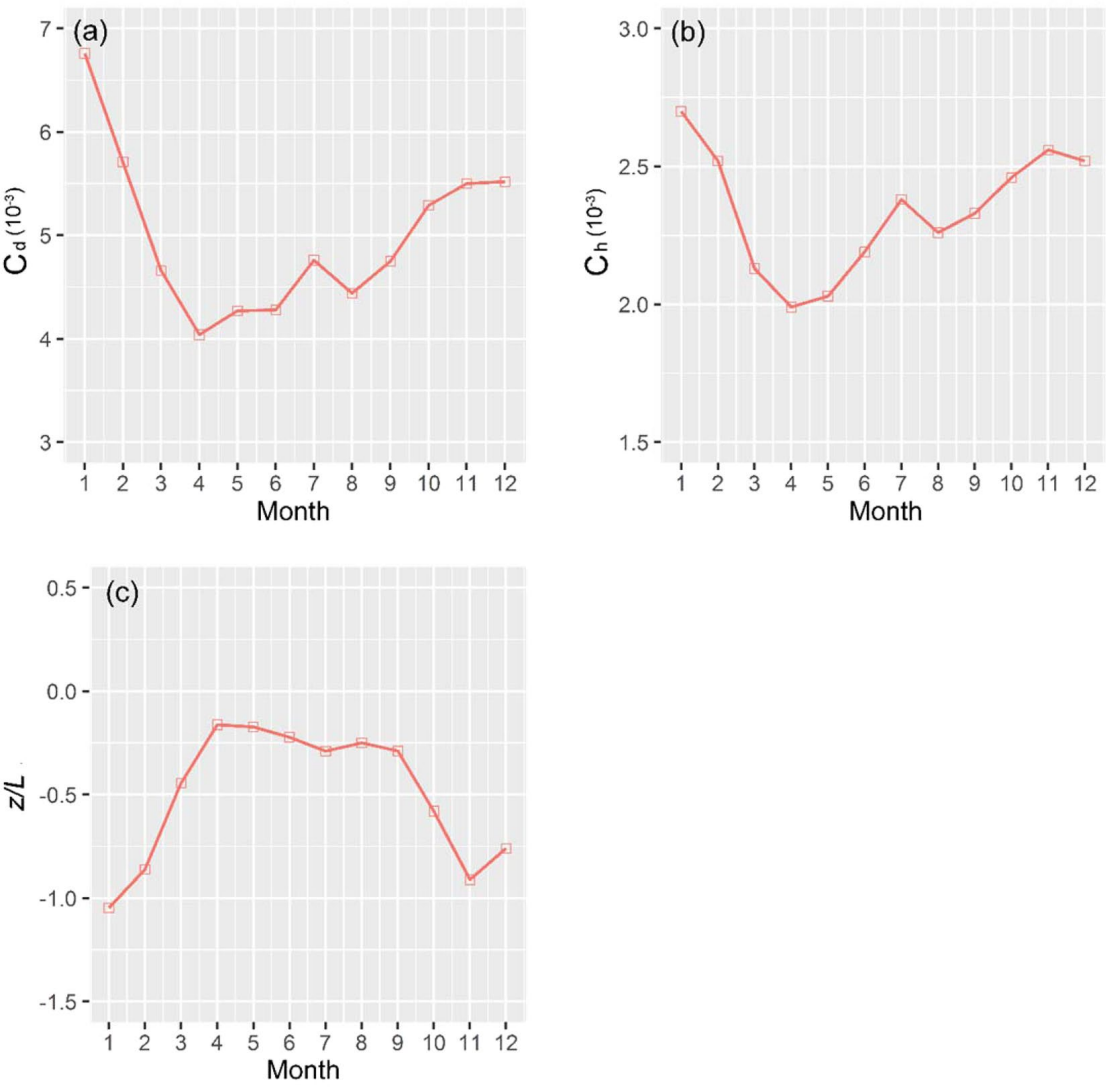
Monthly variations of the means of (**a**) *C*_*d*_, (**b**) *C*_*h*_ and (**c**) atmospheric stability *z*/*L*.

Equation (7) was used to calculate *C*_*d*_ and *C*_*h*_ from the observed fluxes. Their diurnal variations in one year (Fig. 7) and in warm and cold seasons (Fig. 8), their monthly variations (Fig. 9) and relationships with atmospheric stability were also investigated, respectively. From the results, *C*_*d*_ had the mean value of 4.68 × 10^−3^ and *C*_*h*_ had the mean value of 2.26 × 10^−3^. Both were smaller at the station than in the north edge of the TD^20^. The mean value of *C*_*d*_ was larger than in Gobi and other desert areas (e.g., Dunhung and HEIFE)^9,34,35^, but *C*_*h*_ similar to those areas.

**Figure 10:**
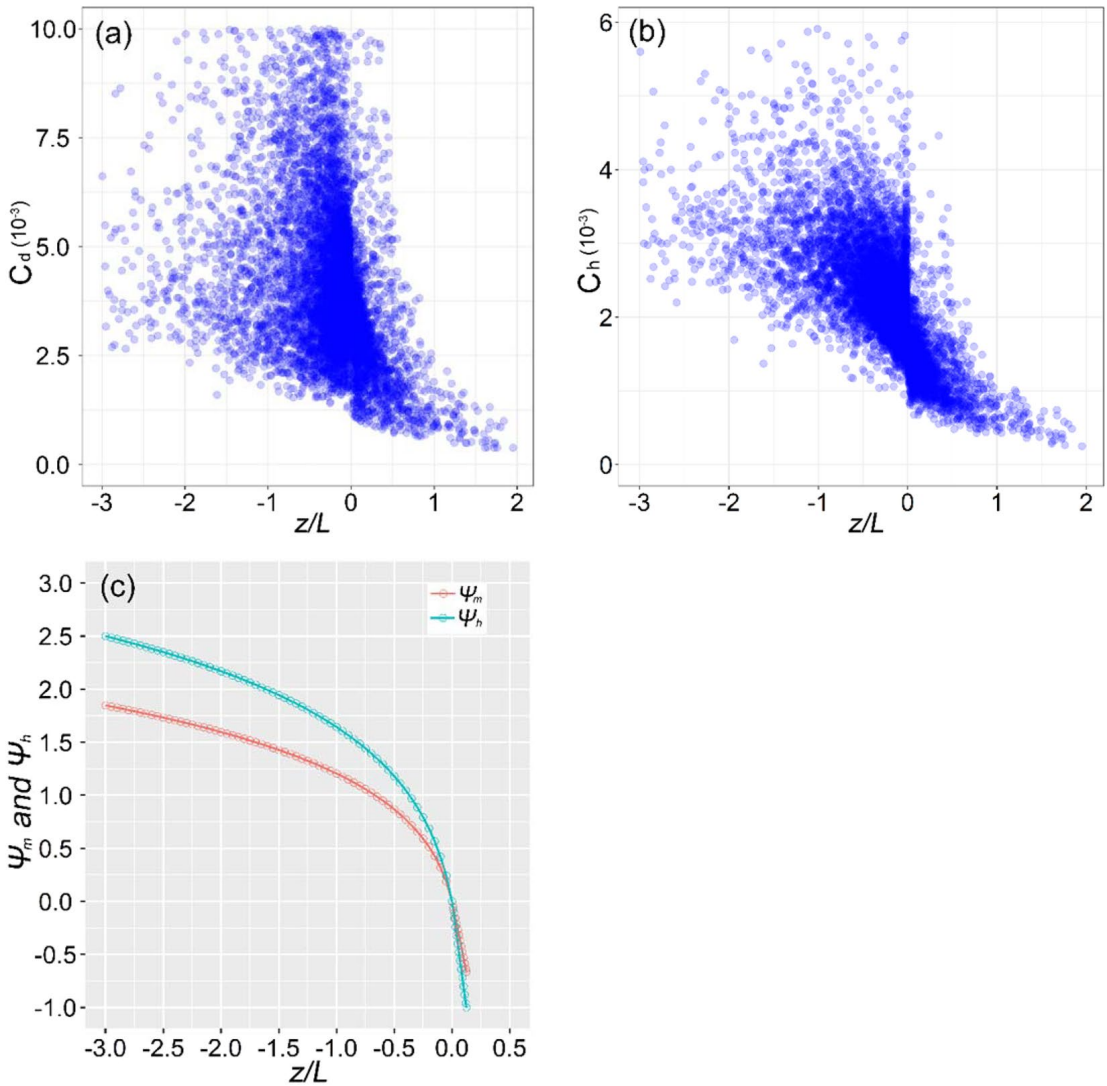
The relationships between (**a**) *C*_*d*_, (**b**) *C*_*h*_ and atmospheric stability *z*/*L*, and (**c**) the variations of the stability functions (*Ψm, Ψh*) and atmospheric stability *z*/*L*.

As is evident in Fig. 7, *C*_*d*_ and *C*_*h*_ are considerably higher during the daytime than the night. When *z*/*L* at the station changed from −0.94 to 0.36, *C*_*d*_ varied from 2.95 × 10^−3^ to 5.81 × 10^−3^ and *C*_*h*_ varied from 1.24 × 10^−3^ to 2.56 × 10^−3^. During the daytime, *C*_*d*_ had the mean value of 5.05 × 10^−3^ and *C*_*h*_ had the mean value of 2.40 × 10^−3^. During the nighttime, the mean values of *C*_*d*_ and *C*_*h*_ were 3.06 × 10^−3^ and 1.31 × 10^−3^, respectively. Both *C*_*d*_ and *C*_*h*_ are inversely correlated with the atmospheric stability, *z*/*L* (Fig. 7c). This result is consistent with the results reported in previous studies for mid-latitudinal areas^25,36,37^. But their peaks do not match closely to the atmospheric stability, because the values are also influenced by surface aerodynamic and thermal roughness lengths. In general, as the unstable surface layer (*z*/*L* < 0) increase, the bulk transfer coefficients will also increase, while as the stable surface layer (*z*/*L* > 0) increase, the bulk transfer coefficients will decrease. Eq. (10) indicates clearly that the bulk transfer coefficients are relate closely to atmospheric stability and surface roughness lengths. According to the significant diurnal variations of roughness lengths (Fig. 5a), two peak values appear at sunrise and sunset, and *z*/*L* changes dramatically from stable (*z*/*L* > 0 at 6:00) to unstable (*z*/*L* < 0 at 7:00) (Fig. 7c, Fig. 8c), and *Ψ*_*m*_(*z*/*L*) and *Ψ*_*h*_(*z*/*L*) exhibit the largest rates of variation, as their values increase rapidly from negative to positive (Fig. 10c). As a result, *C*_*d*_ and *C*_*h*_ also showed diurnal changing patterns, and they responded to changes in the surface roughness lengths and atmospheric stability. Thus, *C*_*d*_ has one peak at sunrise and another highest peak at noon, because the largest value of *z*_0*m*_ appears at sunrise, but the variation of *z*_0*m*_ is small, the highest peak dominated by *z*/*L*. Similar to *C*_*d*_, *C*_*h*_ also has two peaks at sunrise and at noon, respectively, but the highest peak appears at sunrise due to the *z*_0*h*_ has dramatically highest values at sunrise, thus, *z*_0*h*_ dominates the highest peak, and *z*/*L* determines another peak at noon.

We also examined the seasonal variations of *C*_*d*_ and *C*_*h*_, and their relationships with the atmospheric stability. Figure 8 shows that the aforementioned “sunrise effect” is more significant in the cold season than the warm season, because the surface layer at sunrise in the cold season is more unstable than the warm season (Fig. 8c).

**Figure 11:**
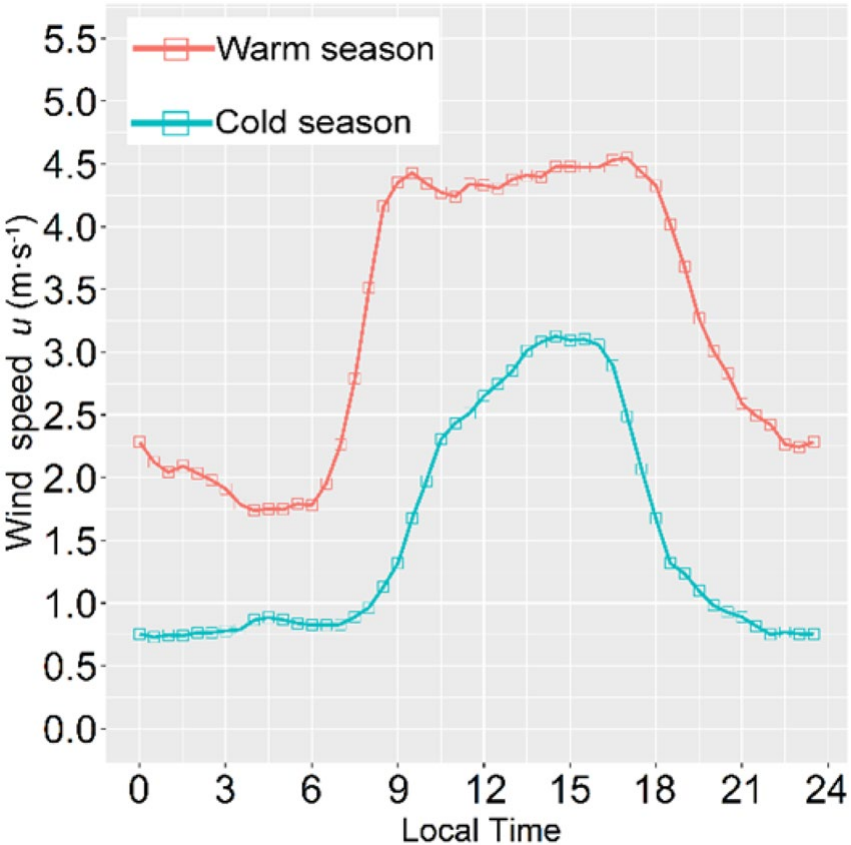
Diurnal variations of wind speed in the warm and cold seasons.

As we known, increased wind speed enhances atmospheric turbulence intensity, consequently decreasing the unstable conditions. The wind speed in the cold season is lower than in the warm season (Fig. 11), which results in increased instability in the cold season.

We note that the “sunrise effect” may be unique for deserts because the works from vegetated areas reveal different temporal patterns^28,37,38^. Both *C*_*d*_ and *C*_*h*_ values in vegetated areas start to rise at sunrise and gradually reach peaks at noon. Another unique feature is that both *C*_*d*_ and *C*_*h*_ values have large values in winter and smaller values in summer because of inverse atmospheric stability (Fig. 9). This phenomenon is nearly completely opposite to that of vegetated areas in southern China^37^, where there are little dust storms happen. *C*_*d*_ varied from 4.08 × 10^−3^ in April to 6.16 × 10^−3^ in January, and *C*_*h*_ varied from 1.99 × 10^−3^ in April to 2.70 × 10^−3^ in January when *z*/*L* ranged from −1.05 in January to −0.16 in April. The variation patterns exhibit extremes in April because the amount of dusts and the number of dust storm days are higher than any other month. This is due to the dramatic interaction of cold and warm air, which enhances the atmospheric turbulence intensity, and decreases the unstable conditions. In addition, if the atmospheric stability is near-neutral, from Eq. (10), *z*/*L* = 0 and *Ψ*_*m*_(*z*/*L*) = *Ψ*_*h*_(*z*/*L*) = 0. Therefore, the surface roughness lengths only determine the bulk transfer coefficients in the near-neutral conditions. Thus, the means of the bulk transfer coefficients were obtained under different conditions (Table 1).

**Table 1:** The annual means of bulk transfer coefficients at different atmospheric stratification^a^.

Bulk transfer coefficients	Stable	Neutral	Unstable	Average
*C*_*d*_ (×10^−3^)	3.01	3.45	5.21	4.68
*C*_*h*_ (×10^−3^)	1.33	1.85	2.55	2.26

^a^Stable (*z*/*L* > 0), Neutral (*z*/*L* = 0) and Unstable (*z*/*L* < 0) represent different atmospheric stabilities, and Average presents the annual mean values.

## Conclusions

Momentum fluxes and sensible heat are two key parameters that characterize the energy exchange process between the land surface and the atmosphere. These parameters can be obtained by calculating the bulk transfer coefficients for momentum (*C*_*d*_) and heat (*C*_*h*_) using the bulk transfer equations. Two important parameters, i.e., the aerodynamic roughness length (*z*_*0m*_) and the thermal roughness length (*z*_*0h*_), need to be determined in the calculation process. Based on the analysis results from the observed data, the following conclusions can be obtained:

1. The aerodynamic roughness length, *z*_*0m*_, in the Taklimakan Desert does not have seasonal variation, instead it is strongly correlated with wind speed. On the contrast, in the vegetated areas, *z*_*0m*_ is greatly dependent on the seasonal variations of vegetation. Therefore, a parameterization scheme of *z*_*0m*_, as it relates to wind speed, is obtained. *z*_*0m*_ decreases at the warm temperature and increases at the cold temperature, with an highest value of 3.11 × 10^−3^ m.
2. The thermal roughness length, *z*_*0h*_, in the Taklimakan Desert is considerably higher during the daytime than at night. On the contrast, in the vegetated areas, *z*_*0h*_ is higher during the night than the day. Two peaks of *z*_*0h*_, i.e., at sunrise and sunset, were observed. These peaks occur when the atmospheric stability reaches near-neutral conditions. The mean value of *z*_*0h*_ is 7.93 × 10^−6^ m.
3. The bulk transfer coefficient values (*C*_*d*_ and *C*_*h*_) have two peaks, one peak appears at sunrise (i.e., so-called “sunrise effect”), due to the instability occurring at sunrise, and the largest roughness lengths for aerodynamic and thermal occurring at sunrise, another peak appears at noon. The “sunrise effect” of the Taklimakan Desert contrasts with the “noon effect” found in vegetated areas, where the values gradually reach their peaks at noon. *C*_*d*_ and *C*_*h*_ had the mean values of 4.68 × 10^−3^ and 2.26 × 10^−3^, respectively.
4. Another unique feature is that both *C*_*d*_ and *C*_*h*_ have larger values in winter and smaller values in summer, being nearly completely opposite to those of vegetated areas. *C*_*d*_ varied from 4.08 × 10^−3^ in April to 6.16 × 10^−3^ in January, and *C*_*h*_ varied from 1.99 × 10^−3^ in April to 2.70 × 10^−3^ in January. In addition, the aforementioned “sunrise effect” is much stronger in the cold season than the warm season, due to the more unstable surface layer at sunrise in the cold season.

Some limitations of this study include that the results are only derived from the Taklimakan Desert. We are not sure whether the characteristics of these parameters agree well with other deserts. However, the topic will be the focus of future studies.

## Data and Methods

### Tazhong station

The Taklimakan Desert is located in the Tarim Depression surrounded by mountains, which is a large internal drainage. The Tianshan Mountains and the inland Tarim River from east to west are located in the north. The Pamir Plateau is located in the west and the Kunlun Mountains are located in the south. The east side of Tarim Depression opens into the Lop Nor Depression. The Taklimakan Desert covers an area of 337,000 km^2^ and is the largest desert in China. It also has the greatest aridity in China. Shifting dunes cover 85% of the total desert, with dunes reaching 20–200 m in height. From the observed data between 1996 and 2013 in the Tazhong Station (Fig. 1a), the annual average temperature is 12.4 °C, the annual average precipitation is only 23.0 mm, and the annual average potential evaporation is 3800.0 mm. The highest temperature in the record is 45.6 °C while the lowest temperature in the record is −32.7 °C. In the summer, the surface temperature can be as high as 80.0 °C. The wind speed has the average value of 2.5 m s^−1^ and with the highest instantaneous value of as high as 24.0 m s^−1^. There are 260 days with sand and dust weather annually.

The data in this paper were collected in the Tazhong Station, which is the only site in the desert hinterland away from the surrounding cities of the Tarim Depression. It is at a linear distance of approximately 300 km from Luntai in the north, 550 km from Sache in the west, 220 km from Niya County in the south and 380 km from Ruoqiang in the east. This unique environment provides good conditions to investigate the atmospheric boundary layer in a desert. Tazhong Station is comprised of a main station (38°58′05″N, 83°39′35″E, altitude 1104 m) and a subsidiary station (38°58′52″N, 83°38′29″E, altitude 1103 m). The main station, located at the oilfield operation area, includes an 80 m gradient detection tower, a three-layer eddy-covariance (EC) system and a radiation observation system. The subsidiary station is located 2.2 km northwest of the main station. The systems in the subsidiary station include an EC system, radiation sensors, soil heat fluxes sensors, and an automatic weather station (AWS). The subsidiary station is located in an open environment with a relatively flat underlying surface in a natural quick sand area. The prevailing wind direction is northeast. The complex sand dunes are present around the subsidiary station, including 850 m in the east, 1600 m in the south and 1700 m in the west. All sand dunes are below 40 m, and run in a northeast-southwest direction, in agreement with the prevailing wind direction. Our study site in this paper is based on the subsidiary station.

### Instrumentation

The main instrumentations at the study site include an eddy-covariance (EC) measurement system, a radiation observation system (Fig. 1b) and an automatic weather station (AWS). In the EC system, a 3D sonic anemometer (CSAT3, Campbell Scientific Inc., USA) is used to measure the velocity and sonic virtual temperature in three dimensions. The EC system is installed 3 m above the ground. The raw data were collected by a CR5000 data logger (Campbell Scientific Inc., USA) at a sampling rate of 10 Hz. The radiation observation system is comprised of four components (CNR-1, Kippp & Zonen, The Netherlands) and used to measure solar radiation and far infrared radiation, which are the downward and upward shortwave and the downward and upward longwave radiation fluxes, respectively. These components were installed at a height of 1.5 m on the same mast as the EC system. The raw radiation data were stored by a CR1000 data logger (Campbell Scientific Inc., USA) at 1 Hz. The AWS, located 30 m northeast of the EC system, was used to measure wind speed/direction profile, air temperature/humidity profile, air pressure, and surface infrared temperature. The AWS data were detected at the sampling rate of 0.1 Hz, and stored by the CR1000 data loggers at 1-minute interval. The instruments are described in details in Table 2. All instrumentations were powered by solar panels and batteries. Raw data were stored on CF cards and output to the laboratory for post-processing every month. The data from January 1 to December 25, 2009 were strictly processed and the average processing time was 30 minutes.

**Table 2:** Observational instruments at the study site.

Observed items	Sensor type	Height (m)
Turbulent fluxes, wind speed, air temperature	CSAT3, Campbell	3
Solar, longwave radiation	CNR-1, Kipp & Zonen	1.5
Air temperature/humidity	HMP45D, Vaisala	0.5, 1, 2, 4
Wind speed/direction	010C and 020C, MetOne	0.5, 1, 2, 4
Surface infrared temperature	IRR-P, Appage	0
Air pressure	PTB220B, Vaisala	1

### Data processing

The post-processing software EdiRe (University of Edinburgh, http://www.geos.ed.ac.uk/abs/research/micromet/EdiRe) was used to acquire the raw data at 10 Hz. Post-processing included spike removal, correction of sonic virtual temperature, the performance estimation of the planar fit coordinate rotation, corrections of density fluctuation (WPL-correction)^39^ and correction of frequency response^40^. In addition, quality control of the half-hour flux data^41^ was conducted based on the following criteria: (1) Data during sensor malfunction (e.g., when there was a faulty diagnostic signal) were rejected; (2) Data within 1 hour window of precipitation were rejected; (3) When the missing data constituted more than 3% of the 30 minutes raw recording, the incomplete 30 minutes data were rejected; (4) Data recorded at the friction velocity below 0.01 m s^−1^ during the nighttime were rejected^42^; and (5) When the wind speed was below 1.0 m s^−1^, and the sensible heat flux had a value below 10 W m^−2^ or the opposite sign to the temperature difference between surface and air (surface minus air), data were rejected. The average value of 30-minute interval radiations was also calculated by removing the out-of-range data. AWS data beyond physical possibilities were rejected. In fact, the AWS data were not used other than as a reference.

## Theory and Methodology

### Surface roughness lengths

Neither *z*_*0m*_ nor *z*_*0h*_ has clear physical meaning. Thus, their values cannot be directly measured. Instead, two methods, i.e., the flux method and profile method, are typically used to estimate *z*_*0m*_ and *z*_*0h*_ from the observed data. In the flux method, the lengths are obtained by applying the bulk transfer equations at a level of observation using eddy-correlation fluxes with specific stability functions. In the profile method, the lengths are obtained from the observed wind and temperature profiles. Both the flux method and the profile method are based on the MOS theory. The integral gradient of the wind and temperature profiles in a horizontally homogeneous surface layer with a stability correction function can be expressed as:

2 $$\mathrm{ln}\,{z}_{0m}=\,\mathrm{ln}(z-d)-\frac{ku}{{u}_{\ast }}-{\psi }_{m}(z/L),$$

3 $$\mathrm{ln}\,{z}_{0h}=\,\mathrm{ln}(z-d)-\frac{k({\theta }_{a}-{\theta }_{0})}{Pr{\theta }_{\ast }}-{\psi }_{h}(z/L),$$

where ${u}_{\ast }$ (m s^−1^), *u* (m s^−1^), ${\theta }_{\ast }$ (K) and *θ*_*a*_ (K) refer to the observed frictional velocity, wind speed, temperature scale and air potential temperature at height *z* (m), respectively. They are known and obtained using the EC system. *K* (with the value of 0.4) is the von Kármán constant, *θ*_*0*_ (K) is surface temperature, *L* (m) is the Obukhov length, *z*/*L* is the dimensionless stability parameter (A negative value means unstable while a positive value means stable) and *Pr* is the turbulent Prandtl number (*Pr* is 1 when *z*/*L* > 0 and 0.95 when *z*/*L* < 0). *Pr* indicates the ratio of the eddy diffusivities between momentum *K*_*m*_ and heat *K*_*h*_, i.e., *Pr* = *K*_*m*_/*K*_*h*_. Because the surface of the Taklimakan Desert is bare, sandy, fairly homogenous and smooth, the displacement height, *d*, is thus negligible, i.e., *d* = 0.

*Ψ*_*m*_(*z*/*L*) and *Ψ*_*h*_(*z*/*L*) are the integrated stability functions for the wind and temperature profiles, respectively. Their formulae were first established based on the data collected from Kansas wheat-farming land by Businger *et al*.^43^. Afterwards, many studies^44–51^ have conducted extensive research on stability functions. It is unknown if those formulae remain valid for the arid surface of the Taklimakan Desert. Thus, the verification and evaluation of empirical stability functions was investigated using the observed data of both flux and gradient. Appropriate *Ψ*_*m*_(*z*/*L*) and *Ψ*_*h*_(*z*/*L*) were determined for stable (*z*/*L* > 0) and unstable (*z*/*L* < 0) surface layers, as given by:

4 $${\psi }_{m}=\{\begin{array}{ll}2\,\mathrm{ln}[(1+x)/2]+\,\mathrm{ln}[(1+{x}^{2})/2]-2{\tan }^{-1}x+\pi /2 & z/L < 0,\\ -5.5z/L, & z/L\ge 0,\end{array}$$

5 $${\psi }_{h}=\{\begin{array}{ll}2\,\mathrm{ln}[(1+y)/2], & z/L < 0,\\ -11z/L, & z/L\ge 0,\end{array}$$

where *x* = (1-12*z*/*L*)^1/4^ and *y* = (1-13*z*/*L*)^1/2^. In many observational studies, the surface temperature is considered to be equal to the surface radiation temperature^27,30,32,37,52,53^. Based on the Stefan-Boltzmann law, the surface radiation temperature, *θ*_*0*_, can be obtained from the upward longwave radiation, ${R}_{lw}^{\uparrow }$(W m^−2^), and the downward longwave radiation, ${R}_{lw}^{\downarrow }$(W m^−2^):

6 $${R}_{lw}^{\uparrow }=(1-{\varepsilon }_{0}){R}_{lw}^{\downarrow }+{\varepsilon }_{0}\sigma {\theta }_{0}^{4},$$

where *σ* (=5.67 × 10^−8^ W m^−2^ K^−4^) is the Stefan-Boltzmann constant, and *ε*_*0*_ is the surface emissivity derived from observations.

From Eq. (2), ln(*z*-*d*) is a variable value on vegetable-covered surfaces due to seasonal changes. For a bare, sandy surface, the value will remain constant. Hence, for bare, sandy surfaces, ln(*z*_*0m*_) is a function of *u*/u_*_ and *Ψ*_*m*_(*z*/*L*). Similarly, from Eq.(3), ln(*z*_*0h*_) is a function of (*θ*_*a*_-*θ*_*0*_)/ θ_*_ and *Ψ*_*h*_(*z*/*L*).

### Bulk transfer coefficients

In the near surface layer, the bulk transfer coefficients are obtained according to the bulk transfer theory, and the momentum and sensible heat fluxes are defined as:

7 $$\{\begin{array}{l}\tau =\rho {C}_{d}{u}^{2},\\ H=\rho {c}_{p}{C}_{h}u({\theta }_{0}-{\theta }_{a}),\end{array}$$

Where *C*_*d*_ and *C*_*h*_ are bulk transfer coefficients for turbulent momentum resistance and heat at the observed level, respectively, *τ* (kg m^−1^ s^−2^) is surface stress, *H* (W m^−2^) is sensible heat flux, *ρ* (kg m^−3^) is the air density, and *c*_*p*_ (with the value of 1004 J kg^−1^ K^−1^) is the specific heat of air at constant pressure. *H*, *τ*, *u*, and *θ*_*a*_ are obtained by the EC system prior to the calculation. In addition, ${u}_{\ast }$ and ${\theta }_{\ast }$ are related to *τ* and *H* by:

8 $$\{\begin{array}{l}\tau =\rho {u}_{\ast }^{2},\\ H=-\,\rho {c}_{p}{u}_{\ast }{\theta }_{\ast },\end{array}$$

From Eqs. (2), (7) and (8), *C*_*d*_ and *C*_*h*_ also can be deduced from MOS theory, and expressed as functions of surface roughness and stability parameters^54^:

9 $$\{\begin{array}{l}{C}_{d}={k}^{2}{[\mathrm{ln}(z/{z}_{0m})-{\psi }_{m}(z/L)]}^{-2},\\ {C}_{h}={k}^{2}P{r}^{-1}{[\mathrm{ln}(z/{z}_{0m})-{\psi }_{m}(z/L)]}^{-1}{[\mathrm{ln}(z/{z}_{0h})-{\psi }_{h}(z/L)]}^{-1}\end{array}$$

## References

[1] Miller RL, Tegen I (1998). Climate response to soil dust aerosols. J. Climate..

[2] Washington R, Todd M, Middleton NJ, Goudie AS (2003). Dust-storm source areas determined by the total ozone monitoring spectrometer and surface observations. Ann. Assoc. Am. Geogr..

[3] Yang B, Bräuning A, Zhang Z, Dong Z, Esper J (2007). Dust storm frequency and its relation to climate changes in Northern China during the past 1000 years. Atmos. Environ..

[4] Xia X (2008). Aerosol optical depth over the Tibetan Plateau and its relation to aerosols over the Taklimakan Desert. Geophys. Res. Lett..

[5] Meng XY, Wang H, Chen J (2019). High-resolution simulation and validation of soil moisture in the arid region of Northwest China. Sci. Rep..

[6] Meng XY (2017). Investigating spatiotemporal changes of the land surface processes in Xinjiang using high-resolution CLM3.5 and CLDAS: Soil temperature. Sci. Rep..

[7] André JC, Goutorbe JP, Perrier A (1986). HAPEX-MOBLIHY: A hydrologic atmospheric experiment for the study of water budget and evaporation flux at the climatic scale. Bull. Am. Meteor. Soc..

[8] Sellers PJ, Hall FG (1992). FIFE in 1992: Results, scientific gains, and future research directions. J. Geophys. Res.: Atmospheres (1984–2012).

[9] Zuo H, Hu Y (1992). The bulk transfer coefficient over desert and Gobi in HEIFE region (in Chinese). Plateau Meteorology.

[10] Bolle HJ (1993). EFEDA- European field experiment in a desertification-threatened area. Annales Geophysicae.

[11] Hall F, Margolis H, Kelly B, Baldocchi D, Wickland DE (1995). The Boreal Ecosystem–Atmosphere Study (BOREAS): An Overview and Early Results from the 1994 Field Year. Bull. Am. Meteorol. Soc..

[12] Lu D (1997). Inner Mongolia semi-arid grassland soil-vegetation-atmosphere interaction (IMGRASS). Global Change News Letter..

[13] Halldin S (1998). NOPEX—a northern hemisphere climate processes land surface experiment. J. Hydrol..

[14] Yamazaki, N., Kalahari, H. & Yatagai., A. Current status of GAME reanalysis project and some preliminary results, In Internation GAME/HUBEX, Workshop Sapporo.12–14 (2000).

[15] Zhang Q, Huang R (2004). Parameters of land-surface processes for Gobi in north-west China. Boundary-Layer Meteorol..

[16] Oncley, S. P. *et al*. The energy balance experiment EBEX-2000. Part I: overview and energy balance. *Boundary-Layer Meteorol.* **123**, 1–28 (2007).

[17] Zhang Q (2009). Some technological and scientific issues about the experimental study of land surface processes in Chinese Loess Plateau (LOPEX) (in Chinese). Advances in earth science..

[18] Liu YQ, He Q, Zhang HS, Mamtimin A (2012). Improving the CoLM in Taklimakan Desert hinterland with accurate key parameters and an appropriate parameterization scheme. Adv. Atmos. Sci..

[19] Liu YQ (2014). Estimation of the land surface emissivity in the hinterland of Taklimakan Desert. J. Mountain Sci..

[20] Jin LL (2018). Observed key surface parameters for characterizing land–atmospheric interactions in the northern marginal zone of the taklimakan desert, china. Atmosphere..

[21] Aynigar Y (2019). Coefficients optimization of the GLASS broadband emissivity based on FTIR and MODIS data over the Taklimakan Desert. Sci. Rep..

[22] Wang MZ (2016). Summer atmospheric boundary layer structure in the hinterland of Taklimakan Desert, China. J. Arid Land..

[23] Chen Y, Yang K, Zhou D, Qin J, Guo X (2010). Improving the Noah land surface model in arid regions with an appropriate parameterization of the thermal roughness length. J. Hydrometeor..

[24] Stull, R. B. An introduction to boundary layer meteorology, pp. 175–177, 383–385, Kluwer Academic Publishers, Dordrecht, Netherlands.(1988).

[25] Garratt JR (1977). Review of drag coefficients over oceans and continents. Mon. Wea. Rev..

[26] Sugita M, Brutsaert W (1990). Regional fluxes from remotely sensed skin temperature and lower boundary layer measurements. Water Resour. Res..

[27] Van den Hurk BJJM, Holtslag AAM (1997). On the bulk parameterization of surface fluxes for various conditions and parameter ranges. Boundary-Layer Meteorol..

[28] Miao M, Ji J (1996). Study on diurnal variation of bulk drag coefficient over different landsurfaces, Meteorol. Atmos. Phys..

[29] Verhoef A, De Bruin HAR, Van den Hurk BJJM (1997). Some practical notes on the parameter kB-1 for sparse vegetation. J. Appl. Meteorol..

[30] Sun JL (1999). Diurnal variations of thermal roughness height over a grassland. Boundary-Layer Meteorol..

[31] Ma Y, Tsukamoto O, Wang J, Ishikawa H, Tamagawa I (2002). Analysis of aerodynamic and thermodynamic parameters on the grassy marshland surface of Tibetan Plateau. Progress in Nature Science..

[32] Yang K (2008). Turbulent flux transfer over bare soil surfaces: Characteristics and parameterization. J. Appl. Meteorol. Climatol..

[33] Wang S, Ma Y (2011). Characteristics of land-atmosphere interaction parameters over the Tibetan Plateau. J. Hydrol..

[34] Zhang Q, Wei GA, Huang RH, Cao XY (2002). Bulk transfer coefficients of the atmospheric momentum and sensible heat over desert and Gobi in arid climate region of Northwest China. Sci. in China (Series D).

[35] Ren HL, Wang CH, Qiu CJ, Dong WJ (2004). A study of computing the surface flux in the typical arid region of northwest China by a variational method (in Chinese). *Chin*. J. Atmos. Sci..

[36] Stull, R. B. Review of non-local mixing in turbulent atmospheres: Transilient turbulence theory. *Boundary-Layer Meteorol.* **62**, 21–96 (1993).

[37] Feng JW, Liu HZ, Wang L, Du Q, Shi LQ (2012). Seasonal and inter-annual variation of surface roughness length and bulk transfer coefficients in a semiarid area. Sci. China Earth Sci..

[38] Liu YQ, Ali M, Meng XY, He. Q (2014). Estimation of the Land Surface Emissivity in the Hinterland of Taklimakan Desert. J. Mt. Sci.-engl..

[39] Webb EK, Pearman GI, Leuning R (1980). Correction of flux measurements for density effects due to heat and water vapour transfer. Quart. J. Roy. Meteorol. Soc..

[40] Moore CJ (1986). Frequency response corrections for eddy correlation systems. Boundary-Layer Meteorol..

[41] Foken, T. *et al* Post-field data quality control, In Handbook of micrometeorology: A guide for surface flux measurement and analysis, edited by X. Lee, W. Massman, & B. Law, pp. 181–208, Kluwer Academic Publishers, Dordrecht, Netherlands (2004).

[42] Blanken PD (1998). Turbulence flux measurements above and below the overstory of a boreal aspen forest. Boundary-Layer Meteorol..

[43] Businger JA, Wyngaard JC, Izumi Y, Bradley EF (1971). Flux-profile relationships in the atmospheric surface layer. J. Atmos. Sci..

[44] Carl DM, Tarbell TC, Panofsky HA (1973). Profiles of wind and temperature from towers over homogeneous terrain. J. Atmos. Sci..

[45] Dyer AJ (1974). A review of flux-profile relationships. Boundary-Layer Meteorol..

[46] Bradley EF, Antonia RA, Chambers AJ (1981). Temperature structure in the atmospheric surface layer. Boundary-Layer Meteorol..

[47] Dyer AJ, Bradley EF (1982). An alternative analysis of flux-gradient relationships at the 1976 ITCE. Boundary-Layer Meteorol..

[48] Högström, U. Non-dimensional wind and temperature profiles in the atmospheric surface layer: A re-evaluation, In Topics in Micrometeorology. A Festschrift for Arch Dyer, pp. 55–78, Springer, Netherlands. (1988).

[49] Högström U (1996). Review of some basic characteristics of the atmospheric surface layer. Boundary-Layer Meteorol..

[50] Frenzen P, Vogel CA (1992). The turbulent kinetic energy budget in the atmospheric surface layer: A review and an experimental reexamination in the field. Boundary-Layer Meteorol..

[51] Zhang Q, Huang R, Tian H (2003). A parameterization scheme of surface turbulent momentum and sensible heat over the Gobi underlying surface. Adv. Atmos. Sci..

[52] Yang K, Tamai N, Koike T (2001). Analytical solution of surface layer similarity equations. J. Appl. Meteorol..

[53] Liu S, Lu L, Mao D, Jia L (2007). Evaluating parameterizations of aerodynamic resistance to heat transfer using field measurements. Hydrol. Earth Syst. Sci..

[54] Garratt, J. R., The Atmospheric Boundary Layer, 316 pp., Cambridge Univ. Press, Cambridge, UK (1992).

